# What about Phenol Formaldehyde (PF) Foam in Modern-Contemporary Art? Insights into the Unaged and Naturally Aged Material by a Multi-Analytical Approach

**DOI:** 10.3390/polym13121964

**Published:** 2021-06-14

**Authors:** Valentina Pintus, Anna Piccolo, Wilfried Vetter, Ligia Maria Moretto, Katja Sterflinger, Manfred Schreiner

**Affiliations:** 1 Institute of Science and Technology in Art, Academy of Fine Arts, Schillerplatz 3, 1010 Vienna, Austria; W.Vetter@akbild.ac.at (W.V.); k.sterflinger@akbild.ac.at (K.S.); m.schreiner@akbild.ac.at (M.S.); 2 Institute for Conservation-Restoration, Modern-Contemporary Art, Academy of Fine Arts Vienna, Schillerplatz 3, 1010 Vienna, Austria; 3 Department of Molecular Sciences and Nanosystems, Ca’ Foscari University of Venice, 30172 Mestre Venice, Italy; piccoloanna98@yahoo.it (A.P.); moretto@unive.it (L.M.M.); 4 Institute of Chemical Technologies and Analytics, Vienna University of Technology, Getreidemarkt 9/164, 1060 Vienna, Austria

**Keywords:** phenol formaldehyde (PF) foam, ageing, optical microscopy, µ-FTIR, pH, modern-contemporary art

## Abstract

The ageing behavior of phenol formaldehyde (PF) foam, a material increasingly used in modern-contemporary art, was investigated by a multi-analytical approach. PF foams with open- and closed-cell structures were selected and analyzed in their unaged and naturally indoor-aged state by employing optical microscopy (OM) and fiber optical reflectance spectroscopy (FORS) for assessing their morphology and color alteration. Micro-Fourier transform infrared spectroscopy (μ-FTIR) was used for determining chemical changes and oxidation processes, and the acidity was monitored by pH measurements. The results clearly showed the extreme sensitivity of both open- and closed-cell PF foams to conditions typically found in indoor museums. OM indicated that the cells of the foams are prone to disrupt, and a tendency towards a red color shift was observed with FORS. μ-FTIR revealed the formation of quinone groups resulting from oxidation reactions. Finally, a slight decrease in the acidity was found by pH measurements.

## 1. Introduction

Phenol formaldehyde (PF) foams have been used in many modern-contemporary artworks in the past, and their utilization is becoming increasingly widespread. Such material undergoes degradation processes quite rapidly because of its intrinsic chemical-physical properties. Therefore, the attention of specialists, such as conservators and conservation scientists, is required in order to understand more about their ageing behavior and to prevent, where possible, their rapid degradation, which can irremediably alter the appearance of the material. 

### 1.1. Phenol Formaldehyde (PF) Foams as a Complex Chemical Formulation

PF foams, characterized by a complex chemical formulation, were first produced in 1937. They are typically prepared by blending, foaming, and curing a phenolic resin with a blowing agent, a surfactant, a curing catalyst, an inorganic filler for regulating their relatively low pH, and other additives, such as colorants and flame retardants [1,2,3,4]. Two different processes can be used in their production, depending upon the desired final product: the continuous or the batch processes. The choice between the two determines the pore structure and thus the percentage of open- and closed-cells in the foam, which is a fundamental parameter that is calibrated depending on desired performance or use [3,5]. To attain specific final properties of the foam, the adjustment of the type and the amount of each component in the formulation is essential.

The phenolic resin is obtained through a condensation reaction between phenol and formaldehyde. When an excess of formaldehyde and a basic catalyst are employed, the so-called resol is formed [2,3,6,7,8]. If a surplus of phenol is made to react with formaldehyde in the presence of an acid catalyst instead, the resulting resin is named novolac [3,5]. The final structure in both cases is characterized by phenolic structures interconnected through methylene and ether bridges and substituted with methylol groups [6]. Phenolic resins can be produced starting from phenol or alternatively cresol and xylenol or crude products of these materials [3]. The selection of the phenols to be used is determinant since the presence of substituted groups governs the reaction outcomes. 

Another component affecting the final product is the blowing agent, which is represented either by volatile liquids or gases dissolved in the polymer or by substances that decompose at high temperatures and release gases, most commonly carbon dioxide. The blowing agent must supply a gas pressure sufficiently high to provide the desired size of cells and at the same time low enough to avoid bursting the cell walls [3,6,9]. The choice of catalyst and curing speed also affects the cell size [3,4,9,10]. While older formulations included corrosive inorganic acids, such as sulfuric acid, which gave the final foam a low pH (ca. 2.5), new phenolic foams have been manufactured by using organic acids, such as p-toluene sulphonic acid and phenol sulphonic acid, which are less corrosive and result in a higher pH (ca. 4–5). Nevertheless, these organic acids are often used in blends with one another and may contain traces of sulfuric or phosphoric acid that were added to speed up the reaction. Further factors in PF foam preparation consist of the type and quantity of surfactant to be implemented, which is fundamental for providing homogeneity in the porosity of the foam [3,4,6,9,10], as well as the kind of additives to be included, e.g., plasticizers, antioxidants, flame retardants, and coloring agents [3,4,5,6,7,9,10]. While pigments and dyes are mainly employed in foam formulations for floral marketing purposes, the other types of additives have technical functions and are used for producing high-quality, closed-cell foams suitable for insulation purposes. These, in fact, are generally more technically difficult to produce and thus more expensive. To have good insulation properties, the foam must be uniform, with a high percentage of closed cells. These trap the blowing gas and are preferably small in diameter to avoid rupture during manufacturing. For obtaining such characteristics, either a high pressure needs to be applied or a specific combination of additives must be used [6,9].

### 1.2. Open- and Closed-Cell PF Foams and Their Applications

Because of their great versatility, PF foams have been employed in many different fields. By adjusting the ratio of open and closed cells in the structure, different characteristics can be attained. The percentage of closed cells is usually about 90% for closed-cell foams and lower than 50% for open-cell ones. In the latter, pores are linked together into a continuous interconnected network, whereas in the former, the individual pores are separated by cell walls. Open-cell foams—normally incorporating a colorant—are produced for floral applications since they can absorb and retain water. Closed-cell foams —generally characterized by the yellow-orange color intrinsic to the polymer—are commonly employed as insulating materials in buildings [3,5,6]. In the art field, thanks to their low rigidity, closed-cell PF foams are employed as a sculptural material since they can be carved easily without the need of sharp tools.

### 1.3. Stability of PF Foams

Most of the objects made from PF foams are meant for everyday use and are durable enough for their intended short service life. However, if these materials are present in artworks or culturally significant artefacts that require preservation, a further study in terms of degradation behavior becomes necessary. Over the long-term, they are likely to show severe problems of ageing and degradation. Foams in general are especially sensitive to oxidation due to their large internal surface area resulting from the many open pores [11]. For this reason, they constitute a critical conservation issue in museum collections. Few studies have been done on the stability of PF foams; most of them focus on the curing and thermal degradation mechanism taking place in phenol formaldehyde resins [7,12,13,14,15,16,17,18]. The thermal degradation mechanism is particularly important since many of these products are marketed as insulating foams. Stable thermal conductivity can be achieved when a closed-cell structure is durable and prevents the exchange of liquids and gasses between the material and its surroundings. The rupture of cell walls and the consistent increase in brittleness result in the loss of some of the material’s properties, compromising thermal insulation. Such phenomena are based on the concurrent entrapment of moisture and air in the foam and gradual outward diffusion of gas used as a blowing agent from inside the foam cells, which is most prevalent around the outer core of the material [15]. When moist air enters the foam, not only are thermal properties altered, but also a chemical change takes place via hydrolysis and/or oxidation. Hydrolysis forms the precursors phenol and formaldehyde, thus causing acidic off-gassing, recognizable by a distinct formaldehyde smell [7]. On the other hand, similarly to the oxidative degradation reaction paths occurring in phenolic formaldehyde resins, oxidation in PF foams may occur. This can be comprised of different simultaneous reactions, but mainly concerns methylene bridges with the consequent formation of benzophenones. Further oxidative degradation leads to the formation of hydroxylated and ketonic species, such as acid fragments and quinones formed by chain scissions [13,14,15,16]. The described reaction sequence is the main path that determines the production of acid compounds. The latter stage of photo-oxidation, during which quinone-type structures are formed, is probably the cause of color change. Because of this, PF foams tend to darken and turn brownish. Such a phenomenon has been observed both in closed- and open-cell types of foams [4,19]. The latter kind of foams, used for floral compositions, are particularly prone to color change not only because the oxidation of the PF polymer is promoted by the ease by which air enters the material, but also because an additional degradation process affects the dyes they contain. It is a requirement of the dyes used for producing the green color to be resistant to acids (for example, organic sulfonic acids commonly used in open-cell foams), otherwise they are not suitable for the composition of the foam. While these dyes, an example of which are triphenylmethane dyes, are compatible with the foaming mixture, they present the disadvantage of being extremely sensitive to light. When their structure is altered, the consequent rapid change in color of the material from green to brown is quickly evident [4].

### 1.4. Aim of This Research

At this time, there is a lack of studies on PF foams that aim to better explain the kinetics behind the degradation processes, such as color alteration, pH change, and increase in brittleness, that consider their complex formulation. Additionally, no studies investigating the pH variations of PF foams in art objects, which could be of help for understanding their degradation mechanisms, have been conducted yet. Based on these considerations, this study aims to investigate the ageing behavior of open- and closed-cell PF foams normally found in modern-contemporary art in indoor conditions by using a multi-analytical approach including optical microscopy (OM), fiber optical reflectance spectroscopy (FORS), micro-Fourier transform infrared spectroscopy (μ-FTIR), and pH measurements. The information obtained by this study will advance the knowledge of an understudied material in modern-contemporary art that is of relevance for many types of professionals, such as conservators, conservation scientists, restorers, and museum curators, and provide them useful information to make meaningful and optimal preservation-conservation decisions.

## 2. Materials and Methods

### 2.1. Materials 

For this work, the following open- and closed-cell PF foams purchased from different manufacturers were selected:

Open-cell PF foam: *Gardol Steckmasse*—n.24787279 (Gardol, Germany); *Oasis Ideal*—n.70-01800 (Oasis, Germany); they both were purchased in March 2020. 

Closed-cell PF foam: *Balsa Foam Soft Density*—n.43016T (AMACO, USA); *Balsa Foam 5 PCF*—n.bf9x6x1-5pcf (American Foam Technologies, Inc., U.S.A.); *Austrotherm Resolution Boden* (Austrotherm, Austria); all three were purchased in October 2018.

These PF foam materials were chosen because of their similarity to the materials that had been recently detected in two studied modern-contemporary artworks [20,21,22].

#### “Unaged” and Naturally Indoor-Aged Materials

In this study, different portions of the selected foams were compared, considering their relatively high sensitivity to ageing and considering their date of purchase. The bulk material, which was sampled from the inner part of the foam after cutting it, will be hereafter referred to unaged with quotation marks as *“Unaged”* and used as reference material, since it is not in direct contact with oxygen and moisture and thus less affected by ageing. The external surfaces will be called *Naturally Aged*. The closed-cell foams, which were purchased in 2018, already presented different colors due to natural ageing. According to this, the *Naturally Aged* closed-cell foams are distinguished as follows:

- Balsa Foam Soft Density: *Naturally Aged 1* is the brighter zone under the label (not glued on the surface), and *Naturally Aged 2* is the one outside the covered area, which resulted in a darker shade.
- Balsa Foam 5 PCF: *Naturally Aged 1* is the portion covered by the label (not glued on the surface), while *Naturally Aged 2* refers to the area surrounding it on the same surface, and *Naturally Aged 3* is the upper lateral part of the block, which was more exposed to the surrounding environment.
- Austrotherm: *Naturally Aged 1* is the surface cut in 2018, and *Naturally Aged 2* is the external one.

The ascending numbers are assigned to the *Naturally Aged* denomination starting from the brightest towards the darkest material (brown) surface areas. 

For floral foams, a distinction between “*Unaged”* and *Naturally Aged* areas was applied, although no differences could be distinguished by the naked eye between the inner and the superficial parts of the foam in terms of color. This is consistent with the fact that they were purchased only a month prior to the analyses for this work. All PF foams were stored on a laboratory table in indoor conditions that ranged from ca. 20 °C and 40% RH of the winter period (October to April) to a maximum of ca. 24 °C and 55 % RH in summer (May to September). Moreover, the foam materials were subjected to night-day light variations through daylight lamps with a correlated color temperature (CCT) of 5000 K.

### 2.2. Optical Microscopy (OM)

To document and characterize the selected foams in terms of surface morphology and structure, such as cell type and diameter, samples were observed through optical microscopy (OM). Two different types of optical light microscopes were employed: an Axioplan 2 Imaging microscope (10×, 20×, and 50× objectives and 10× oculars) (ZEISS, Germany) was employed with visible reflected (Vis) and UV lights (UV lamp HBO 100). The images of the sample were acquired by a Nikon D700 Camera and evaluated with the Camera Control Pro2 Software. The other was a Keyence VHX-6000 digital microscope (RZ 100x-1000x objective—VH-Z100R Keyence, Japan) with a LED (light emitting diode) light source characterized by a color temperature of 5700 K. This optical microscope was provided with an LCD monitor, and the images were acquired by a CMOS camera equipped with an 1/1.8-inch CMOS image sensor (virtual pixels: 1600 (H) × 1200 (V)). 

### 2.3. Fiber Optics Reflectance Spectroscopy (FORS) 

Information about the color of the selected PF foams surface was derived by fiber optics reflectance spectroscopy (FORS) analyses. These were performed through a spectrophotometer MSP 400 (J&M, Germany) equipped with a 256-diode array detector, which allows measurements in the region from 300–1150 nm. The optical system consists of the built-in halogen lamp of the spectrometer. Two quartz fiber optic cables direct the beam from the source to the measuring point on the object (1250 μm core diameter, length 2 m) and from the object to the spectrometer (600 μm core diameter, length 2 m). The geometry of measurements was 0°/45°. Working in diffuse reflectance by collecting the light scattered at 45° with respect to the incident light allowed avoiding specular reflected light. Spectra were acquired in the wavelength range from 360 to 1000 nm with an integration time of 100 milliseconds, and 5 scans were averaged for each spectrum. As a white reference, the Zenith Polymer target sphere optics calibrated over the UV-Vis-NIR range (diffuse full material target, reflectivity 99%, item number SG 3110) was used. For each sample, at least 10 measurement points were chosen and analyzed. The reflectance spectra obtained were collected with the software TIDASDAQ 2.35 (J&M, Germany) and evaluated with the software Panorama 2.1 (LabCognition, Analytical Software GmbH, Germany). For the natural indoor ageing studies, the total color values (ΔE*) were obtained according to the Commission Internationale de l’Eclairage (CIE) 1976.

### 2.4. Micro-Fourier Transform Infrared (μ-FTIR) Spectroscopy 

Micro-Fourier transform infrared (μ-FTIR) analyses were performed with a LUMOS standalone FTIR microscope (Bruker Optics, Germany) equipped with a Globar thermal light source, a RockSolid interferometer, and a liquid nitrogen cooled mid-band 100 × 100 μm^2^ photoconductive mercury cadmium telluride (PC-MCT) detector. Transmission, reflectance, and attenuated total reflection (ATR) mode were tested on the first sample of foam to determine the best method of investigation. The ATR probe was a germanium frustum cone-shaped crystal (Ge, refractive index *n* = 4) with a tip diameter of 100 μm. This ATR probe is incorporated into a fully motorized and automated piezo motors 8x Cassegrain objective (NA = 0.6). A XYZ motorized sample stage allows selecting a priori the applied pressure of the ATR probe during the measurements in three different modes, such as low, medium, and high. Due to the brittleness of the foams, a low pressure was selected. However, even this left an imprint on the sample and did not provide satisfactory spectra. All the optics and beam-splitters are made of zinc selenide (ZnSe). Spectra were acquired in the spectral range between 4000 and 600 cm^−1^, performing 128 scans at a 4 cm^−1^ resolution. Transmission mode was preferred for the measurements, since this method required only a small sample (less than 1 mm in diameter) to be taken and provided high resolved spectra with a high signal-to-noise ratio, unlike the reflection and ATR modes. For the analyses in transmission, tiny amounts of sample material were taken from the foams and prepared on a diamond anvil compression cell (SPECTRA TECH, Shelton, USA) using a syringe needle. After pressing the sample between the two cell windows, one of those windows with no remnant of the sample was removed, while the other one containing the well-pressed sample was placed on the XYZ motorized sample stage for the analyses. The background acquisition was taken with one cell window. For each sample, at least 5 measurement points with 100 × 100 µm^2^ were chosen and analyzed. The resulting spectra were collected and evaluated with the spectrum software OPUS-IRTM Version 8.0 (Bruker Optics, Germany). 

### 2.5. pH Measurements

The pH measurements were carried out by the suspension technique [10]. This method was performed by first pulverizing 0.05 g of foam in a jade mortar and mixing it with 20 mL of distilled water inside a sealed flask. This was left stirring for 7 days on a magnetic stirrer plate whose speed was kept constant at 800 rpm. The pH of the suspension was finally tested on PF foams triplicates with a portable LAQUAtwin B-172 pH meter (Horiba, Germany) of small dimension (164 mm × 29 mm × 20 mm). Equipped with electrodes on one side and a digital LCD-screen on the other side of the length, this pH meter allows the pH measurements of tiny drops (0.1 mL) —to be placed in contact with the electrodes—in a relatively short time (approx. 15 s) and with high precision (pH ± 0.1). The pH measurement range is between 2 and 12.

## 3. Results and Discussion

The terminology used for referring to the selected PF foams will be as follows: “floral foams” will mean the open-cell Gardol and Oasis foams, “sculpting foams” will refer to the closed-cell Balsa foam soft density and Balsa foam 5 PCF types, and “insulating foam” will denote the closed-cell Austrotherm foam. 

### 3.1. Optical Microscopy (OM)

The main characteristics of the phenol formaldehyde (PF) foams studied by optical microscopy (OM) are summarized in Table 1. 

#### 3.1.1. “Unaged” Open- and Closed-Cell PF Foams

**Figure 1:**
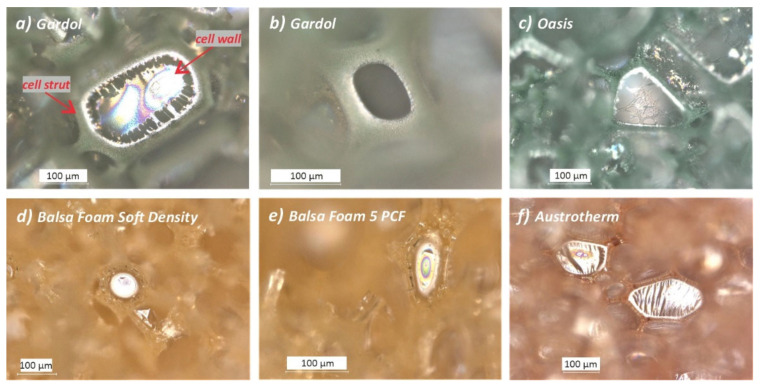
Photomicrographs of different polygonal shapes of cells observed in the *“Unaged”* Gardol (**a,b**), Oasis (**c**), Balsa foam soft density (**d**), Balsa foam 5 PCF (**e**), and Austrotherm (**f**) foams by digital optical microscope with reflected light.

By definition, foams are characterized by pores or cells with membranes between them called cell walls, while the cell-wall intersections rich in PF resin are named cell struts or ribs; these form a complex network structure (Figure 1). 

**Figure 2:**
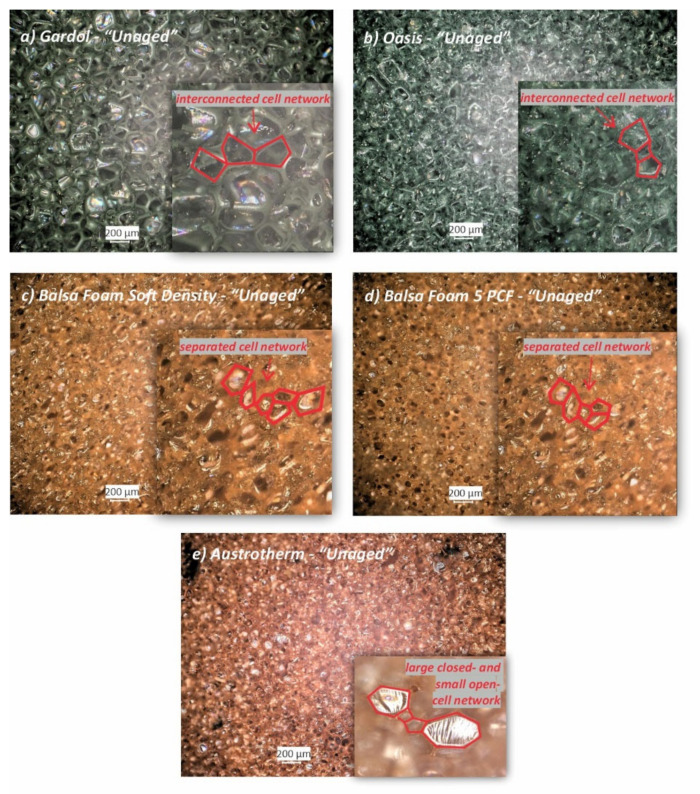
Photomicrographs of the *“Unaged”* phenol formaldehyde (PF) foams (**a**–**e**) investigated by a digital microscope with reflected light.

By observing the *“Unaged”* PF foams with the OM, it was possible to first appraise some fundamental differences between the open- and closed-cell PF foams, which are listed and described here: 

*Cell network*: as shown in Figure 2a,b, in floral PF foams, the pores were mostly linked together into a continuous interconnected network while showing more heterogenous cell structures, whereas in the sculpting PF foams (Figure 2c,d), the individual pores or cells were not linked but separate from one another. Unlike the sculpting foams, the insulating foam had many connected, adjacent, large closed and small open cells, probably indicating a higher percentage of closed cells in the foam (Figure 2e). 

*Cell wall*: the cell walls, which appear as thin iridescent layers connecting the sides of the polygonal shapes (Figure 2), were mostly destroyed in the case of the open-cell PF foams, which showed remarkably bigger diameters and with a wider range of diameters variations in comparison to the closed-cell PF ones (Table 1). 

*Cell shape*: another difference which was assessed was the variability of the cell’s’ polygonal shapes in terms of number of sides. As shown in Figure 1 and reported in Table 1, while floral and sculpting foams were mostly made up of pentagonal and hexagonal cells, respectively, heterogeneity was observed in the insulating foam, where cells ranged from four to seven sides (Figure 1f). Although few cases of four- and six-sided cells were observed (as shown in Figure 1a,b), Gardol foam had mostly pentagonal cells with two sides being longer than the others (Figure 2a), whereas the open-cell Oasis showed a higher percentage of cells having similar lengths for all the five sides of the polygon (Figure 2b), although some cells presented two sides shorter (Figure 1c). 

**Figure 3:**
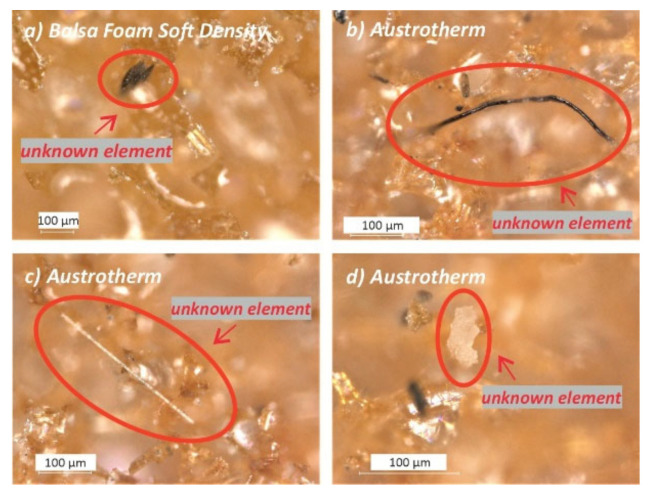
Photomicrographs of some unknown elements observed in the *“Unaged”* Balsa foam soft density (**a**) and in Austrotherm (**b**–**d**) foams by a digital microscope with reflected light.

*Cell struts*: the cell struts of closed-cell PF foams appeared to be more complex in terms of composition compared to open-cell ones. In the first case, struts are less smooth, presenting small bubbles, granules, filaments, and thin black outlines. Moreover, different kinds of unknown elements were detected in such foams, some examples of which are shown in Figure 3a–d (red circles). 

**Figure 4:**
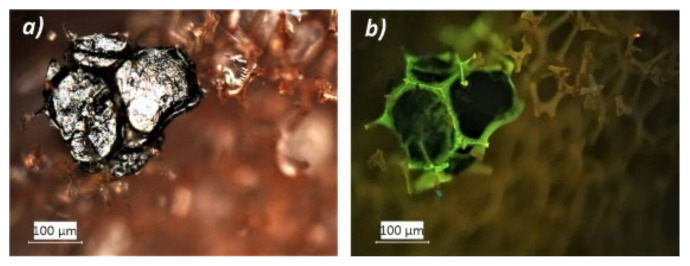
Photomicrographs of an unknown element detected in Austrotherm foam in reflected visible (**a**) and UV light (**b**) by optical microscopy.

These are likely fibers and other additives that were added in the production mixture of closed-cell PF foams to prevent cells from bursting and to obtain longer lasting materials. These characteristics are important especially in insulating materials, in which thermal properties are intended to persist over time. Compared to the other analyzed foams, indeed, the insulating foam was observed to contain the highest amounts of unknown elements. Many of these were fluorescent when irradiated by UV light (Figure 4a,b).

#### 3.1.2. Naturally Aged Open- and Closed-Cell PF Foams 

The ageing of the selected PF foams by exposure to indoor environmental conditions had great effect on the material structure. This was mostly observed to a larger extent on the upper surface of the closed-cell PF foams, which were purchased two years prior to the analyses, than the open-cell PF ones, which were acquired two months before the investigations. By comparing the *“Unaged”* and *Naturally Aged* PF foams, two main aspects were observed in some of the selected materials and are described below. 

**Figure 5:**
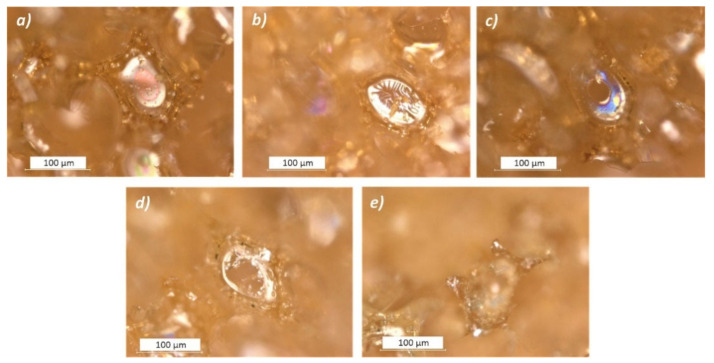
Proposed phases of the cell-rupture mechanism observed by a digital microscope with reflected light: (**a**) bubbles increase and get bigger; (**b**) a circular mark appears on the thin layer of the cell; (**c**) a hole is formed; (**d**) the layer breaks; and (**e**) cell walls become thinner and brittle.

*(i) Rupture of the cell walls with width reduction of cell struts*: the sculpting foams showed thinner cell struts in the aged parts, indicating that a width reduction phenomenon had occurred and was likely the cause of the rupture of most of the cell walls. Only the smallest cells remained unspoiled. For example, while for the “*Unaged”* Balsa foam 5 PCF an average strut thickness of ca. 21 µm was calculated, for the *Naturally Aged 1, 2,* and *3*, it was ca. 12 µm. Considering the change in size, shape, and appearance assessed in the cells through the microscopic observations, a common mechanism for cell rupture due to natural ageing can be proposed. An example of this is shown in Figure 5: (a) the original cell wall looks like marked with a circle, (b) then it gets thinner and thinner due to an elongation of the cell until it ruptures, and a hole appears in its center. Afterwards, (c) the cell wall starts presenting many cuts, while bubbles inside the cell struts increase in number and size. This process leads to (d) the depletion of the cell walls, which in the end break, causing the whole structure to collapse. These processes were more widespread the higher the level of the *Naturally Aged* denomination (from *1* to *3*), which probably determined the brittleness of the material.

On the other hand, the insulating foam was observed to be more stable in terms of structure. In both the “*Unaged”* and the *Naturally Aged* parts of the material, no cell wall ruptures or altered cell strut widths were detected. This higher stability in comparison to the other PF foams may be attributed to a larger number of unknown elements used as additives in the cell structure, which prevented cell rupture, resulting in a longer lasting material. In the floral foams, cell wall rupture was observed in naturally aged foams after just two months, although not as widely as in the sculpting foams. The open-cell floral foam already had holes in most cell walls before ageing. This characteristic likely leads to prompt breaking, caused by the collapse of the strands that connect the cell struts. Moreover, it was observed that the phenomenon of the distortion of the polygonal shape of cells due to the depletion of ribs was even more widespread in floral foams.

**Figure 6:**
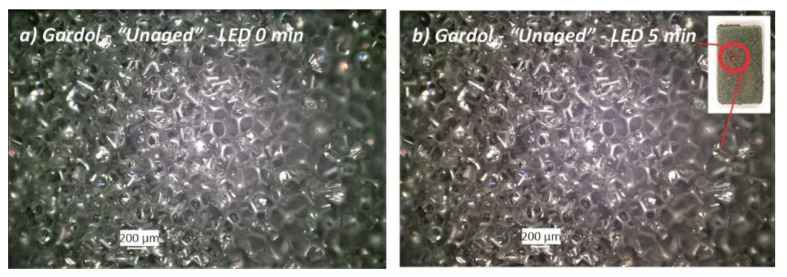
Open-cell *“Unaged”* Gardol foam sample before (**a**) and (**b**) after five minutes of LED illumination of the optical microscope used for the observation and documentation of the materials. Brown areas caused by the LED illumination are shown in (**b**) and circled in red in the foam sample image on the top right.

*(ii) Sensitivity to the light of the optical microscopes*: the open-cell Gardol foam was found to be extremely sensitive to the lights of the optical microscopes employed for the materials observation and documentation. For instance, as it can be observed in Figure 6: the foam turned from green to brown after five minutes of exposure to the digital microscope LED lamp. This fading of the green color is evidence of its high photosensitivity; the brown coloration is typical of phenol formaldehyde resin. Nevertheless, OM revealed no significant changes in the cell-rupture condition. In contrast to what was observed in Gardol foam, the open-cell Oasis foam, which also has a green coloration, proved to be less sensitive to the microscopes’ illumination, since no color changes were observed after exposure to the light of both microscopes. This may be due to different types of colorant used for the floral foams, which may be of interest for a subject of further research since the chemical nature of the green colorants used for both floral foams remained undetected and unknown within this study. 

### 3.2. Fiber Optics Reflectance Spectroscopy (FORS)

The results of FORS measurements for all the investigated PF foams are summarized in Table 2, where the shift in the values of the lightness/darkness (L*), redness/greenness (a*), yellowness/blueness (b*), and the total color difference (ΔE*) are also reported. 

Due to the lack of studies on PF foams, particularly in the field of art conservation, FORS data references for this type of material were not available yet. Therefore, the recognition of the coloring agent in the investigated PF foams with these analyses was extremely challenging. Furthermore, pigments and dyes are not specified by the manufacturers of the products. Nevertheless, the registered reflectance spectra and colorimetric data were useful for comparing the *“Unaged”* and *Naturally Aged* foams and thus for detecting the changes that occurred on the surface. 

#### 3.2.1. “Unaged” Open- and Closed-Cell PF Foams

**Figure 7:**
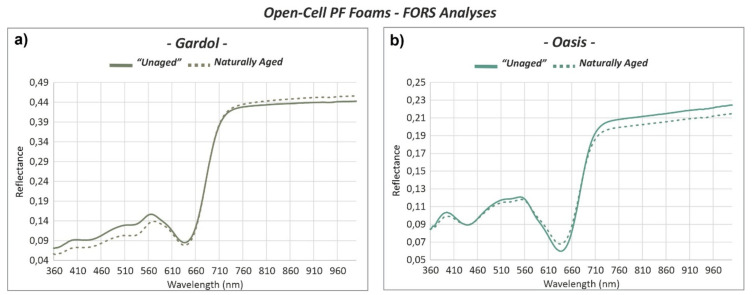
FORS reflectance spectra of the *“Unaged”* and *Naturally Aged* (**a**) Gardol and (**b**) Oasis open-cell PF foams.

Reflectance spectra registered for the two green “*Unaged”* floral foams showed some characteristic bands in the region between 400 and 700 nm (Figure 7a,b). While “*Unaged”* Gardol had three main reflectance maxima, the most prominent at 567 nm, likely related to its yellow component that causes the overall olive-green color, and two weaker maxima at 510 and 405 nm. “*Unaged”* Oasis showed a higher one at ca. 559 nm, with a weak one at 512 nm and an additional maximum at 390 nm. The latter indicates the major contribution of blue/violet component of the color, which generally appears as jade green. 

The stronger contribution of the yellow component in Gardol is shown by the greater b* (12.3) value in comparison to Oasis (3.0), which, on the other hand, has a major green input, according to a* = −25.5 versus a* = −8.0 of Gardol (Table 2). A noticeable difference between the reflectance spectra of Gardol and Oasis foams can be seen between 700 and 1000 nm: although they both appeared flat, Oasis had a lowest reflection value of 0.21, while that of Gardol was 0.43. This is likely due to a different roughness of the surface. Such differences in the reflectance spectra of the two floral foams are likely ascribable to the different chemical nature of their green colorants, which also would support their contrasting photosensitivity observed under the OM. 

**Figure 8:**
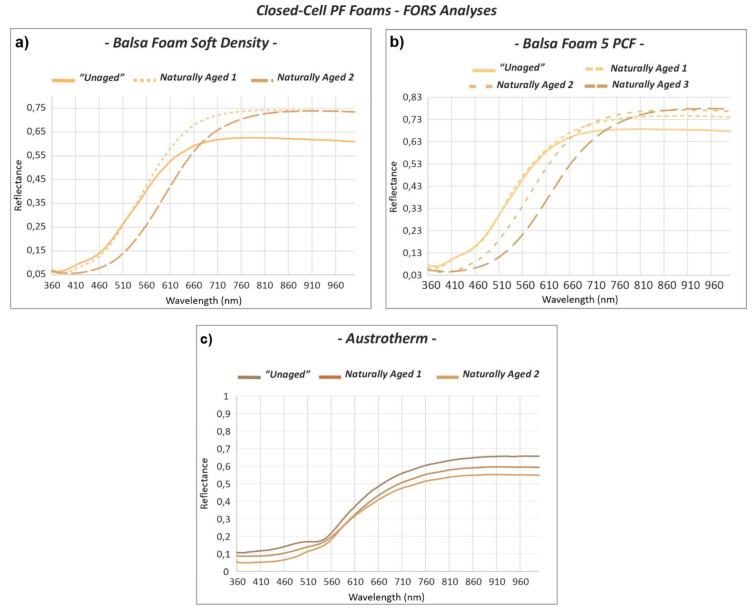
FORS reflectance spectra of the *“Unaged”* and *Naturally Aged* (**a**) Balsa foam soft density, (**b**) Balsa foam 5 PCF, and (**c**) Austrotherm closed-cell PF foams.

As it was possible to anticipate by looking at their coloration, the reflectance spectra of the yellow-orange “*Unaged”* sculpting (Figure 8a,b) and insulating (Figure 8c) foams were very different from those of the green floral foams.

They are mostly characterized by a gradually increasing curve in reflectance from 400 to 680 nm, which then reaches a plateau at higher wavelengths. Only one weak maximum can be found at around 400 nm for the two sculpting foams, while for the insulating foam, an absorption band is present at ca. 510 nm. The latter material also presented a wider absorption range up to 550 nm in comparison to the narrower absorption range of the sculpting foams, ranging up to 460 nm. This difference is complemented by the colorimetric parameters of the yellow-orange sculpting foams corresponding to approx. L* = 84, a* = 19, and b* = 50 and of the pale pink insulating foam of ca. L* = 59, a* = 23, and b* = 25. The higher b* value in the sculpting foams clearly indicates their greater yellow contribution with respect to the insulating foam. 

#### 3.2.2. Naturally Aged Open- and Closed-Cell PF Foams

**Figure 9:**
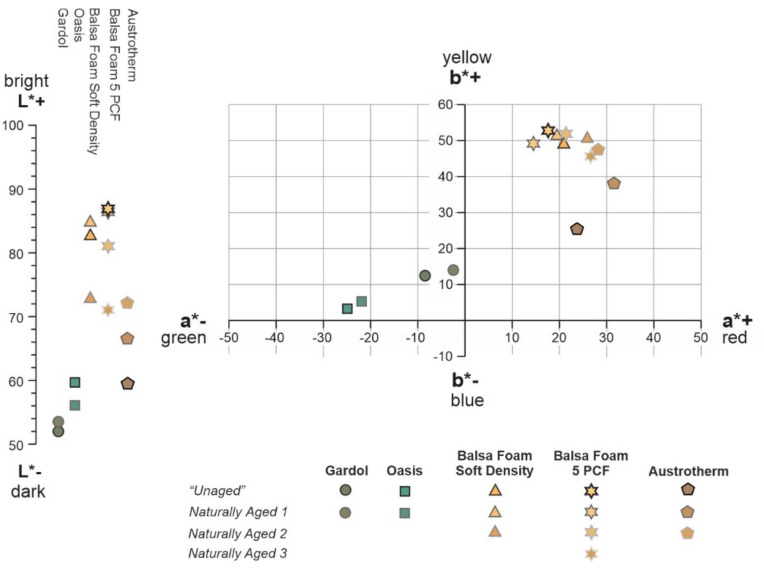
CIELab diagram of the open-cell Oasis and Gardol PF foams as well as of the closed-cell Balsa foam soft density, Balsa foam 5 PCF, and Austrotherm PF foams showing the shifts of their L*, a*, and b* coordinates.

The CIELab diagram in Figure 9 reports the occurrence of color changes, highlighting the shift of a* and b* values after natural ageing. On the left side of the figure, the difference in L* values corresponding to the brightening and darkening of the foam’s surfaces is shown. 

*Naturally Aged* open- and closed-cell PF foams were easily distinguishable by the naked eye from the “*Unaged”,* since their color was much darker with hues towards brown. This observation was confirmed by the obtained ΔE* values (Table 2), ranging from ca. 3 to 6 for the open-cell foams and from 1 to 18 for the closed-cell ones. According to the literature [23], an unexperienced observer would notice differences in color for a total color shift (ΔE*) above 2. The collected values clearly highlight the ease by which PF foams undergo ageing phenomena, as evident by color changes. These mainly consist of the great shifts of a*, indicating an increase in the red contribution. Moreover, the low reflectance values registered in the blue and violet as well as in the near UV portions of the reflectance spectra further explain these color changes. Indeed, such regions of the electromagnetic spectrum (such as near UV, which was absorbed by all the investigated samples) contain the necessary energy to cleave the chemical bonds. Generally, the closed-cell foams exhibit greater differences in the reflectance spectra and more pronounced shifts of the L*, a*, and b* coordinates between the “*Unaged”* and *Natural Aged* materials than the open-cell ones. This result is principally related to the longer ageing period of two years for the closed-cell foams in contrast to the one of two months for the open-cell foams.

By comparing the spectra between the “*Unaged”* and *Naturally Aged* parts of the open-cell PF foams (Figure 7a,b), a reflectance decrease was in the region between 360 and 640 nm after ageing. In the Gardol foam, a small increase in reflectivity could be observed starting from ca. 700 nm (Figure 7b). In the Oasis foam, a decrease was registered at the same range, with also a slight increase between 600–680 nm (Figure 7a). Moreover, a slight change from green to yellow/red shades with ageing is attested to the Gardol and Oasis foams by both the shift of the reflectance maximum at 566 nm towards higher wavelengths and the increase of b* and a* values. The yellow and red components become more prominent in both Gardol and Oasis foams as ∆b* = 1.9 and 1.7 and ∆a* = 5.6 and 3.3, respectively. 

A common trend was observed for the *Natural Aged* sculpting foams (Figure 8a,b): the higher the *Natural Aged* denomination, the stronger the shift of the absorption band towards higher wavelengths. Furthermore, the reflectance values in the region below 650 nm tended to become lower, while the ones from approx. 700 nm tended to rise. This indicates a change in hue towards red, as it is also attested to by the meaningful increase of a* to much higher values (Table 2). Additionally, a decrease in L* corresponding to a darkening of the sculpting foam surfaces was determined. Likewise, in the insulating foam, a shift towards higher wavelengths and an increase of a* showing a change towards red (∆a* = 7.1) was observed for the *Naturally Aged* area but in a lesser extent with respect to the other closed-cell foams. In contrast to the other foams, a decrease in reflectance was registered in the whole spectral range of the *Naturally Aged* areas of the insulating foam (Figure 8c). The diversity in the increase and decrease in reflectance values registered for the *Naturally Aged* PF foams from 700 nm on originates from physical or scattering differences. As it was noticed through the optical microscope, the surface of the naturally aged open-cell Gardol and sculpting closed-cell foams had more irregularities than the “*Unaged”*, such as a greater amount of cell ruptures and microbubbles in the cell struts. This is probably the cause of the higher reflectance values detected for the naturally aged zones of the sample compared to the unaged ones in the region between ca. 700 to 1000 nm. The naturally aged open-cell Oasis and closed-cell insulating foam, which according to the OM were characterized by fewer irregularities and higher stability, presented slightly lower reflectance values from 700 nm on.

### 3.3. Micro-Fourier Transform Infrared (μ-FTIR) Spectroscopy

The µ-FTIR absorption bands of both “*Unaged”* open- and closed-cell PF foams are summarized in Table 3, while the main changes observed on the *Naturally Aged* PF foams are reported in Table 4. The assignment of the relevant bands was done on the basis of reference literature mostly focused on PF resins and foams [5,8,13,24,25,26,27,28,29,30,31,32,33,34,35,36,37,38,39].

#### 3.3.1. “Unaged” Open- and Closed-Cell PF Foams

The µ-FTIR measurements performed on the *“Unaged”* open- and closed-cell PF foams revealed the main characteristic IR bands of phenol formaldehyde, resulting from the contribution of phenolic and methylene groups and their linkages. These bands can be distinguished in different main groups, such as phenolic O-H and C-O, aromatic C=C and C-H, methylene groups CH_2_, ether bridges C-O-C and methylol groups CH_2_OH, and additional vibration as oxidation groups C=O, aromatic C=C, and methylene groups CH_2_.

**Figure 10:**
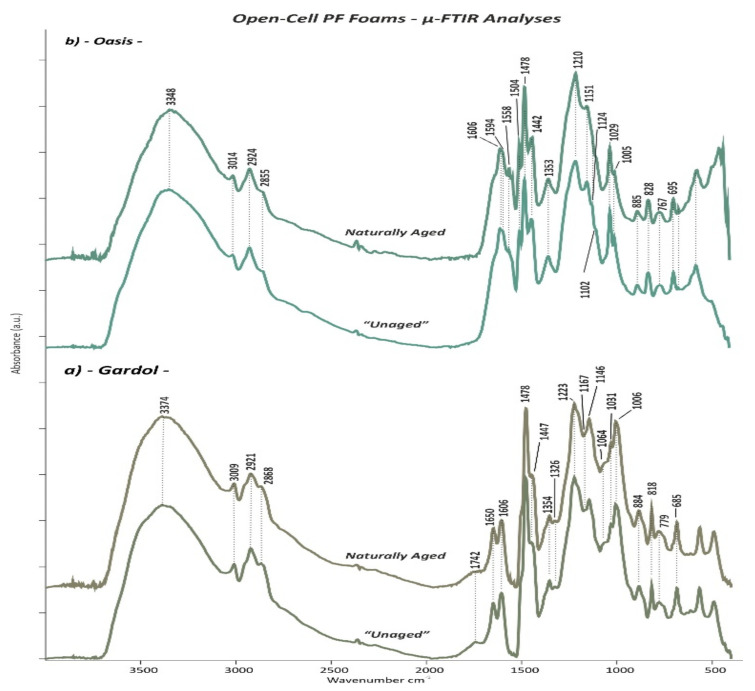
µ-FTIR spectra of the *“Unaged”* and *Naturally Aged* (**a**) Gardol and (**b**) Oasis open-cell PF foams.

**Figure 11:**
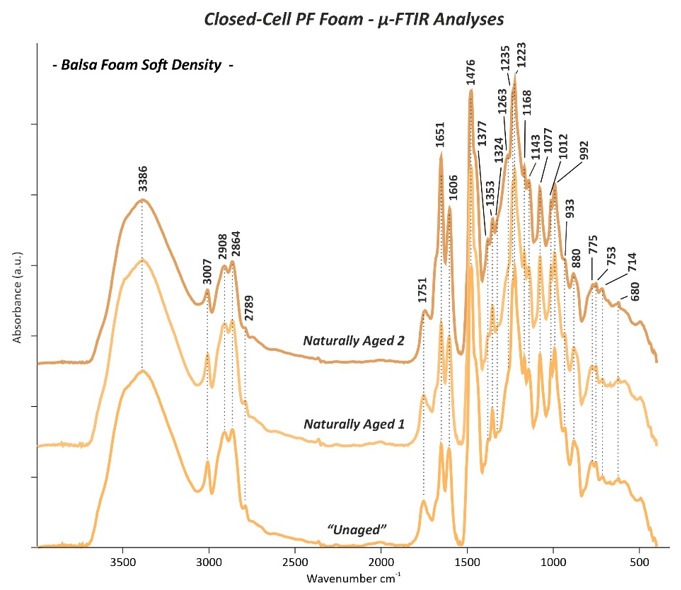
µ-FTIR spectra of the *“Unaged”* and *Naturally Aged 1* and *2* Balsa foam soft density closed-cell PF foam.

**Figure 12:**
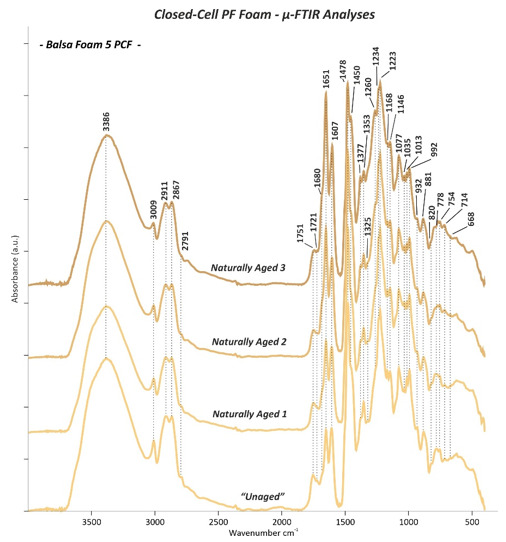
µ-FTIR spectra of the *“Unaged”* and *Naturally Aged 1, 2*, and *3* Balsa foam 5 PCF closed-cell PF foam.

**Figure 13:**
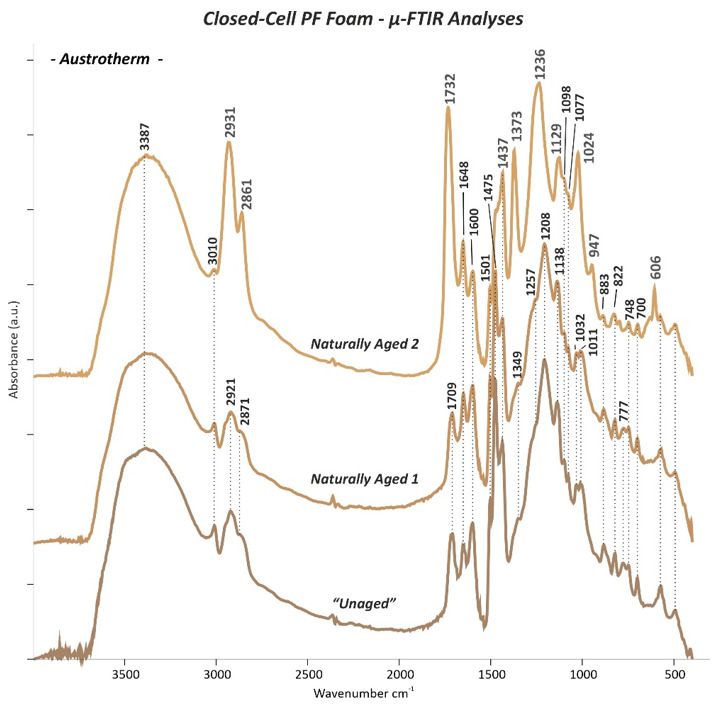
µ-FTIR spectra of the *“Unaged”* and *Naturally Aged 1* and *2* Austrotherm closed-cell PF foam. Wavenumbers highlighted in grey in the *Naturally Aged 2* Austrotherm indicate the typical bands of a PVAc coating.

*Phenolic OH and C-O*: the presence of the hydroxyl groups of phenols was indicated by the O-H stretching as a broad and strong band between 3100 and 4000 cm^−1^ [5,8,24,25,26,27,28] with a maximum in the range from 3387 and 3348 cm^−1^ for the all investigated “*Unaged”* PF foams (Figure 10, Figure 11, Figure 12 and Figure 13). The in-plane bending of the same functional group resulted in a peak at ca. 1353 cm^−1^ [5,8,24,25,26,28]. Additionally, a strong and intense band given by the phenolic C-O stretching between 1223 and 1208 cm^−1^ was detected [5,24,25,26,27,28,30]. A further contribution of the latter was determined at ca. 1235 cm^−1^ as a less intense peak in the IR spectra of the closed-cell foams (Figure 11, Figure 12 and Figure 13). This band has been assigned in the literature also to the C-O-C ether bonds [27]. 

*Aromatic C-H and C=C*: the stretching of aromatic C-H bonds was detected as a small band between 3007 cm^−1^ and 3014 cm^−1^ in all PF foams [5,24,27,29]. This band was complemented by the aromatic on-plane bending C-H in the region between 1168 and 1064 cm^−1^ [5,8,24,26,28] and the out-of-plane bending C-H in the range from 885 to 680 cm^−1^ [5,13,24,25,26,28,30,31]. Many peaks were registered in this last interval, since different types of substituted aromatic rings can be present in the PF foams. More precisely, C-H out-of-plane deformation in the monosubstituted benzene ring was registered as a band between 680 and 700 cm^−1^. The peak between 748 and 767 cm^−1^ corresponded to a phenol with di- substitutions at 1,2 positions (*o*: ortho position), while at around 779 cm^−1^, it was given by a phenol with tri-substitutions at 1,2,6 positions (*o,o’*: ortho-ortho position). The peak between 818 and 826 cm^−1^ is attributable to phenol with di-substitutions at 1,4 positions (*p*: para position), and the 882 cm^−1^ peak was identified as phenol with tri- and tetra-substitutions at 1,2,4 (*o,p:* ortho-para position) and 1,2,4,6 positions (*o,o’,p*: ortho-ortho-para position), respectively. Some IR peaks related to the C=C stretching in aromatic rings could also be detected at 1606 cm^−1^ [5,8,24,29]. This vibrational C=C stretching mode was found to have a further contribution at 1594 and 1558 cm^−1^ [5,8,24,25,26,28,29] for the open-cell Oasis foam (Figure 10b). At 1503 cm^−1^, the IR spectra of both the open-cell Oasis and closed-cell Austrotherm foams presented a distinctive sharp peak, while for the open-cell Gardol and the sculpting closed-cell foams, only a weak shoulder could be identified. Such discrepancies are probably due to the differences in terms of the position of substituents in phenolic rings of the open- and closed-cell PF foams and likely related to the phenol formaldehyde molar ratio used for the foam production.

*Methylene groups CH_2_:* methylene groups, which connect aromatic rings, were detected by the aliphatic CH_2_ stretching signals as two bands between 2921 and 2864 cm^−1^ in all the analysed foams and a further smaller peak at ca. 2798 cm^−1^ for the closed-cell ones [5,24,25,27,28]. The presence of such groups was confirmed by a sharp doublet with the most intense peak at 1472 cm^−1^ (*o-p’*: ortho-para position) and a less intense one at 1447 cm^−1^ (*p-p’*: para-para position) due to C-H scissor bending [31,32,33]. This last peak also masks the stretching C=C aromatic contribution occurring in the same position [28,29] and the CH_3_ aliphatic stretching band at 1437 cm^−1^ [27] occurring in the spectrum of the closed-cell insulating foam (Figure 13). An additional contribution of the methylene groups was registered in the IR spectra of the open-cell Gardol (Figure 10a) and sculpting closed-cell foams (Figure 11 and Figure 12) as a tiny overtone peak at 1326 cm^−1^ attributable to C-H bending.

*Ether bridges C-O-C and methylol groups CH_2_OH:* in all samples, methylene ether bridges existing between aromatic rings could be detected by the aromatic C-H in-plane deformation ranges between 1151–1138 cm^−1^ and 1098-1064 cm^−1^, due to the stretching of C-O-C bonds [5,27,28,29]. The presence of methylol groups (CH_2_OH) is discernible from the peaks of C-O stretching between 1036 and 1006 cm^−1^ [5,24,25,26,27,28,29], while the O-H stretching band is obscured by the broad and strong band of the O-H phenolic groups in the region between 3387 and 3348 cm^−1^ [5,24,26,29]. 

*Additional vibrations as oxidation groups C=O, aromatic C=C, and methylene groups CH_2_*: the majority of the IR spectra acquired from the “*Unaged”* PF foams showed some very noticeable IR bands in the range between 1750 and 1645 cm^−1^. Among those, the most intense one was detected at ca. 1650 cm^−1^, which is caused by C=O stretching. This is likely ascribable either to the carbonyl group of a benzophenone structure arising from the oxidation of methylene bridges [13,27] and/or to formaldehyde, present because of hydrolysis or as the residue of the incomplete reaction with phenol [8]. Considering the first hypothesis as the most plausible, it could be supposed that a large number of methylene bridges had undergone oxidation already during the production of the PF foam. Additionally, a tiny peak at around 1721 cm^−1^ was found in the sculpting closed-cell foams (Figure 11 and Figure 12), which suggests that oxidation processes occurring in methylene groups had progressed, leading to chain scission and to the formation of hydroxy acid groups COOH [13]. 

According to the µ-FTIR results obtained in this study, the intensity of the carbonyl band at 1650 cm^−1^ was inversely proportional in intensity to the aromatic C=C peak at 1501 cm^−1^ and to the C-H bending at 1447 cm^−1^ (*p-p’*: para-para position) of the methylene groups CH_2_. For instance, while the open-cell Oasis did not show a band at 1650 cm^−1^ but instead sharp peaks at 1501 and 1447 cm^−1^ (Figure 10b), the closed-cell Balsa foam 5 PCF generated a very intense band at 1650 cm^−1^ and a shoulder at 1447 cm^−1^ but no band at 1501 cm^−1^ (Figure 12). 

As has been demonstrated by Manfredi et al. [31], the intensity of such bands and their variation are related to the formaldehyde-to-phenol (F/Ph) molar ratios. By increasing the F/Ph ratio from 1.2 to 1.6, the carbonyl band at approx. 1650 cm^−1^ is formed, while the bands at ca. 1501 cm^−1^ and 1447 cm^−1^ lose their intensity, indicating a lower phenol content and higher degree of methylene groups oxidation, respectively. For a F/Ph ratio of 2.0 and 2.5, the band at 1501 cm^−1^ is no longer detectable, indicating a lower phenol content, while the band at 1650 cm^−1^ increases. Additionally, the increase of F/Ph ratio corresponds to an increase of the quantity of tri-substituted phenol compounds with the IR band at 779 cm^−1^ because of the higher degree of conversions [31]. Considering these observations and according to the IR spectra obtained for the “*Unaged”* PF foams in this study, it is possible to distinguish the analysed samples in terms of F/Ph ratios in the following order: *closed-cell Balsa foam soft density>closed-cell Balsa foam 5 PCF>open-cell Gardol>closed-cell Austrotherm>open-cell Oasis.*

Furthermore, the band at 1650 cm^−1^ was directly proportional in intensity to the C=O stretching shoulder detected at 1685 cm^−1^, which confirms the high level of oxidation, since the latter is related to the formation of quinoid type structures [13]. These bands were also directly proportional in intensity to the peak at 1751 cm^−1^—registered in the sculpting closed-cell foams (Figure 11 and Figure 12) —and to the broad band at 1742 cm^−1^ of the open-cell Gardol (Figure 10a). Both signals occur in the 1755–1735 cm^−1^ range, which is associated with the C=O stretching of aryl carboxylic acid monomers [35] and not of the associated form placed between 1735 and 1650 cm^−1^. 

The frequency of the bond vibration is influenced by the surrounding molecular structure such as the nature of the constituent R group [36] and, more specifically, by its electronegativity [37]. The carbonyl stretching bond shifts to lower wavenumbers by decreasing the bond force constant when the dipolar character is increased [36]. In the literature, the assignment of the 1742 cm^−1^ peak in phenolic resin has some contradictions; it has been related either to carbonyl or carboxylic groups formed through oxidation reactions [35,38] or to tetra-substituted benzene rings [27,36]. Other vibrations, which may be associated to aryl carboxylic acids, were observed for the sculpting closed-cell foams (Figure 11 and Figure 12) at circa 1377 (O-H in plane bending), 1260 (C-O stretching), 992 (=C-H bending), and 933 cm^−1^ (O-H out of plane bending) [38,39]. On the other hand, the band at 1751 cm^−1^ was not found in previous studies of PF foams. Unfortunately, the complexity of the PF foams formulation and the few studies existing on their identification and stability make the assignment of IR band variations with certainty challenging. In order to clarify the assignment of these bands registered in the C=O carbon-acids region and also to better elucidate the chemical formulation of such complex materials, supplementary analyses are necessary (e.g., Py-GC/MS). These will be the subject of a near future publication.

#### 3.3.2. Naturally Aged Open- and Closed-Cell PF Foams

Comparing the IR spectra between the “*Unaged”* and *Naturally Aged* open- and closed-cell PF foams, some differences in the absorption bands can be observed. These were most prominent for the sculpting foams (*Naturally Aged 2* Balsa foam soft density, Figure 11 and *Naturally Aged 3* Balsa foam 5 PCF, Figure 12), while the floral foams seemed to be more stable. Such evidence is consistent with the fact that closed-cell PF foams were purchased two years before the measurements for this study instead of the two months of the open-cell foams. 

**Figure 14:**
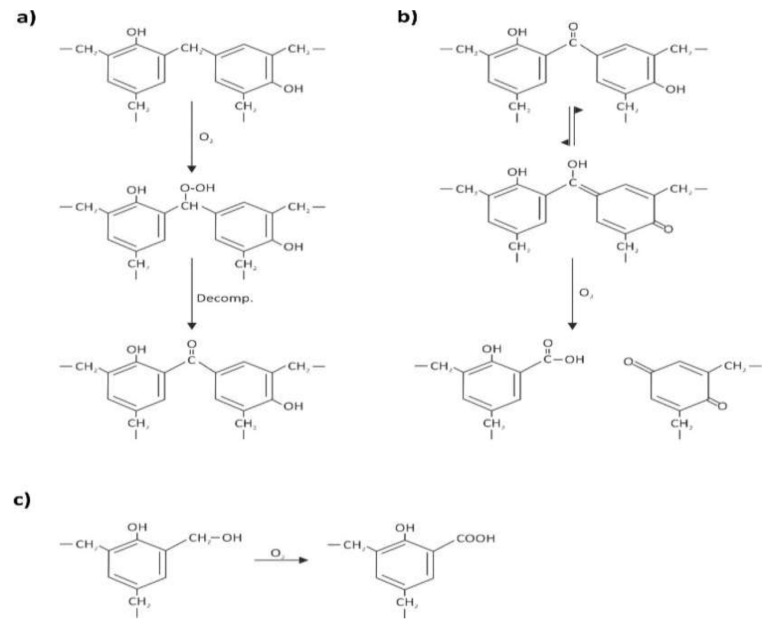
Mechanisms of oxidation reactions on phenol formaldehyde (PF) after Conley [13]: (**a**) primary photo-oxidation reactions: oxidation at a methylene linkage resulting in the formation of carbonyl groups; (**b**) secondary photo-oxidation reactions: formation of quinone-type structures; and (**c**) oxidation of methylol groups and formation of acidic moieties.

The recorded changes in the IR spectra are listed in Table 4 and can be tentatively explained by the two main stages of oxidation reactions proposed by Conley [13]: 

(1) Primary oxidation reactions are based on the oxidation of the methylene linkages resulting in the formation of carbonyl groups of, e.g., benzophenone linkages (Figure 14a) and responsible for the increase in absorption of the C=O band at 1650 cm^−1^ and decrease in intensity of the CH_2_ methylene bridge bands at 1479 cm^−1^ and particularly at 1447 and 1326 cm^−1^. The ketonic group band at 1650 cm^−1^ greatly surpassed in intensity the nearby C=C aromatic signal at 1601 cm^−1^, especially in case of the sculpting foams. These variations in intensity were much more pronounced for the samples which theoretically had a higher F/Ph ratio, such as sculpting foams (Figure 11 and Figure 12) and open-cell Gardol (Figure 10a). 

(2) Further, secondary oxidation reactions took place in the investigated PF foams, especially in the sculpting closed-cell ones, by involving chain scissions of quinoid linkages (Figure 14b). This is evidenced by the increase in intensity of carbonyl IR bands such as the shoulder at 1680 cm^−1^, due to the formation of quinone-type structures, and by the higher intensity of the band at ca. 1721 cm^−1^ related to hydroxy acids fragments, as shown in Figure 12. This is complemented by the weakening of the phenolic hydroxyl band at around 1354 cm^−1^. Quinones are mostly considered responsible for the color shift towards brown evidenced in the naturally aged PF foams by FORS measurements. Additionally, oxidation reactions involved the methylol groups CH_2_OH (Figure 14c), determining a loss in intensity of the C-O stretching bands between 1005 and 1035 cm^−1^ and the likely consequent formation of acidic components, e.g., salicylic acids and related hydroxybenzoic acids [12]. 

The strong C=O stretching peak at 1751 cm^−1^ of aryl carboxylic acid detected in the “*Unaged”* sculpting foams was observed to shift to ca. 1744 cm^−1^ with ageing (Figure 11 and Figure 12). This shift towards lower wavenumbers is likely due to a modification in the surrounding molecular structure and its electronegativity [36,37]. This change was complemented by an increase in intensity of the peaks at 1377 (O-H in plane bending) and 1260 cm^−1^ (C-O stretching) of the acid groups. 

Great differences could be found comparing the spectra obtained for the inner (“*Unaged”* and *Naturally Aged 1*) and the outer (*Naturally Aged 2*) parts of the thermal insulating foam (Figure 13). By examining such IR spectra, it was possible to detect the presence of an additional component to the foam: ethylene-vinyl acetate (EVA), probably used as a coating material. Distinctive IR bands of an EVA are given by the C=O at 1732 cm^−1^; C-H stretching at 2931 and 2861 cm^−1^; C-O stretching at 1236, 1127, and 1024 cm^−1^; C-O and C-C stretching at 947 cm^−1^; and CH_2_ rocking at 606 cm^−1^ [40,41,42,43,44].

### 3.4. pH Measurements 

The suspension method used for measuring the pH of the “*Unaged”* PF foams showed that the floral foams Gardol and Oasis had a similar pH value, around 2.9 (Table 5), close to the one of insulating foam Austrotherm. 

On the other hand, the sculpting foams Balsa foam soft density and Balsa foam 5 PCF had slightly higher pH values, 3.80 and 4.00, respectively. By comparing the pH values obtained from the *“Unaged”* and *Naturally Aged* PF foams, it was evidenced that only a nearly negligible decrease in terms of acidity could be noticed after two months of natural, indoor ageing for the floral foams and after two years of natural ageing for the sculpting and insulating foams. More precisely, the slightest variation of about ± 0.1 was found for the floral open-cell Gardol and Oasis and the insulating closed-cell Austrotherm, while the biggest variation of ± 0.2–± 0.3 was found for the sculpting closed-cell foams. Considering that the insulating foam was subjected to a natural indoor ageing for two years similarly to the sculpting foams while the floral foams were aged for two months, this result may demonstrate the higher stability of the insulating one in respect to the floral foams but also to the sculpting foams. Since the acidity of the PF foams is predominately related to the type of inorganic and/or organic acids used as catalyst in the chemical formulation, which can remain in traces in the final product, the slight decrease in acidity registered by the pH measurements may indicate their chemical variation during the natural indoor ageing. Further investigations for determining the chemical nature of the catalysts and their variation upon ageing (e.g., by Py-GC/MS) may be beneficial for better explaining the decrease in terms of acidity of the PF foams. These will be also the subject of a near future publication.

## 4. Conclusions

A multi-analytical approach was used for investigating open- and closed-cell phenol formaldehyde (PF) foams—often used in modern-contemporary art—in their unaged state as well as after natural ageing in indoor conditions. 

Firstly, meaningful differences in the structure, chemical composition, and pH of open- and closed-cell foams were verified. OM showed that the open-cell floral foams had quite irregular-shaped cells with large diameters and mainly no walls. The closed-cell sculpting and insulating foams appeared more regular in structure and had a smaller diameter but were characterized by a higher complexity in composition confirmed by the presence of unknown elements. Furthermore, while the open-cell floral foams and the closed-cell insulating foam had an acidic pH value (around 3), the sculpting closed-cell foams had a higher pH (approaching 4). Finally, µ-FTIR analyses showed, based on the dissimilar IR band intensities, positions, and type found, that the foams were slightly different in their chemical composition with variations in terms of formaldehyde-to-phenol (F/Ph) molar ratios.

The high instability of PF foams and the difference between open- and closed-cell foams in terms of color variation, brittleness, and chemical changes upon ageing in indoor conditions were attested within this study. More precisely, the color variation towards brown in all PF foams observed under naked eye—more noticeable for the closed-cell sculpting foams—corresponded to the shift towards higher wavelengths reported in the FORS spectra and the significant shift in the a* color coordinate towards red. OM showed the rupture of the cell walls with width reduction of cell struts resulting from ageing for the closed-cell sculpting foams. This change is the likely cause of their brittleness. Not only did the optical appearance of the PF foams changed with ageing, but their chemical structure did as well. The presence and increase of benzophenone type bridges, arising from the oxidation of methylene ether groups, and of quinone and hydroxy acids formed as a result of main chain cleavage was demonstrated by µ-FTIR spectral data. Those results showed that primary and secondary oxidation reactions took place on the materials and especially on the sculpting closed-cell foams. Furthermore, a gradual and slight increase in pH to a more alkaline value was observed for the naturally aged open- and closed-cell foams in comparison to the unaged ones. Based on the obtained results within this study, particular care should be taken for modern-contemporary objects made of PF foams, which clearly showed a strong tendency to color variation and brittleness. The coexistence and interaction of various organic compounds within the PF foams define their overall chemical composition and their color and pH variations. Further research for elucidating the chemical formulation of such complex materials and for specifying trends and correlating factors acting in the indoor ageing processes would certainly expand knowledge on the topic and assist in the development of optimal conservation guidelines for PF artifacts and artworks. 

## Figures and Tables

**Table 1:** List of the phenol formaldehyde (PF) foams investigated, distinguished according to their cell type, commercial name, application, color, cell structure characteristics, and general observations summarized after natural ageing (two months for open-cell foams and two years closed-cell foams).

*Structure Cell Type*	*Commercial Name*	*Application*	*Color*	*Cell Diameter (µm)*			*Cell Struts Thickness (µm)*			*Cell Shape*	*General Observations after Natural Ageing*
				*Min*	*Max*	*Average*	*Min*	*Max*	*Average*		
Open-cell	Gardol	floral arrangments		86	272	181	16	39	28	polygonal from 4 to 6 sides (mainly 5)	cell walls rupture
	Oasis			109	224	180	13	22	18	pentagonal (few with 2 sides shorter)	elongations of cells
Closed-cell	Balsa Foam Soft Density	sculpting		100	158	129	16	27	23	hexagonal	browning/elongation of two sides cells/cell walls rupture/brittle cell struts
	Balsa Foam 5 PCF			78	130	97	14	30	21	hexagonal	browning/cell walls rupture/brittle cell struts
	Austrotherm	thermal insulation		61	122	88	7	16	11	polygonal from 4 to 7 sides	Browning/brittle cell struts

**Table 2:** L*, a*, b* as CIELab color coordinates obtained by FORS analyses on the *“Unaged”* and open- and closed-cell PF foams. Their shift and the total color change (∆E*) are also reported.

*Structure Cell Type*	*Samples*		*Color*	*CIELab Color Coordinates*			*CIELab Color Shifts*			*Total Color Change*
				L*	a*	b*	∆L*	∆a*	∆b*	∆E*_1976_
Open-cell	Gardol	“Unaged”		52.4	−8.0	12.3	1.1	5.6	1.9	6.0
		Natually Aged		53.5	−2.4	14.2				
	Oasis	“Unaged”		59.2	−25.5	3.0	−2.3	3.3	1.7	4.4
		Natually Aged		56.9	−22.2	4.7				
Closed-cell	Balsa Foam Soft Density	“Unaged”		82.4	20.6	49.8	2.1	−1.6	1.1	2.9
		Naturally Aged 1		84.5	19.0	50.9				
		Naturally Aged 2		73.6	26.3	49.3	−10.9	7.3	−1.6	13.2
	Balsa Foam 5 PCF	“Unaged”		86.8	17.3	51.1	−0.6	−1.9	−2.1	2.9
		Naturally Aged 1		86.2	15.4	49.0				
		Naturally Aged 2		80.3	21.2	50.1	−5.9	5.8	1.1	8.3
		Naturally Aged 3		70.5	26.4	45.9	−9.8	5.2	−4.2	11.9
	Austrotherm	“Unaged”		59.1	23.2	25.0	7.1	7.4	14.8	18.0
		Naturally Aged 1		66.2	30.6	39.8				
		Naturally Aged 2		72.1	28.5	47.1	5.9	-2.1	7.3	9.6

**Table 3:** µ-FTIR absorptions of the open- and closed-cell *“Unaged”* phenol formaldehyde (PF) foams investigated and their assignment according to the literature data [5,8,13,24,25,26,27,28,29,30,31,32,33,34,35,36,37,38,39].

PF Foams							
	Open-Cell		Closed-Cell				
Bond Type	Gardol	Oasis	Balsa Foam Soft Density	Balsa Foam 5 PCF	Austrotherm	Lit. Data Range [5,8,13,24,25,26,27,28,29,30,31,32,33,34,35,36,37,38,39]	Lit. Compound Type Assignment
	Wavenumber cm^−1^						
O–H stretching	3374	3348	3386	3386	3387	3389–3100	Phenolic O–H [5,8,24,25,26,27,28] and methylol OH [5,24,26,29]
C–H stretching	3009	3014	3007	3009	3010	3300–3010	Phenolic ring C–H [5,24,27,28]
	2921; 2868	2924; 2855	2908; 2864	2911; 2867	2921; 2871	2953–2800	Methylene group –CH_2_– [5,24,25,27,28]
	O	O	2789	2791	O	2724	CH_2_ groups of formaldehyde (–CHO) [25]
C=O stretching and C–C stretching	1742.000	O	1751.000	1751.000	O	1755–1735	Arlyl carboxylic acid [35,38]/Tetra–substituted benzene ring C–C stretching [27,36]
C=O stretching	O	O	1722 (shoulder)	1721	O	1720	Hydroxy acids COOH [13]
	O	O	O	O	1709	1704	(Phenolic) C=O [34]
	O	O	1685 (shoulder)	O	O	1660–1690	Quinoid structures [13]
	1650	O	1651	1651	1653; 1648	1643	Formaldehyde monomer residue [8]/benzophenone by oxidation of methylene groups [13,27]/(Phenolic) C=O [34]
C=C stretching	1606	1606; 1594; 1558; 1504	1606; 1503 (weak shoulder)	1607; 1503 (weak shoulder)	1600; 1501	1633–1500	Phenolic ring C=C [5,8,24,25,26,28,29]
C–H bending and C=C stretching	1478	1478	1476	1478	1475	1480–1473	Scissor bending vibration of CH_2_ *(o–p’ )* [31,32,33]
	1447	1442	1450 (shoulder)	1450 (shoulder)	1451 (weak shoulder)	1456–1450	Scissor bending vibration of CH_2_ *(p–p’)* [31,32,33] and C=C aromatic ring [28,29]
	O	O	O	O	1436	1437	Aliphatic CH_3_ [27]
O–H in plane bending and C–H bending	O	O	1377	1377	O	1378–1370	Phenolic O–H groups [5,24,25] /C–H deformation vibration of aliphatic hydrocarbons [27] / O–H in plane bending of carboxylic acids [39]
	1354	1353	1353	1353	1349	1360 –1340	Phenolic O–H groups [5,8,24,25,26,28] / C–H deformation vibration of aliphatic hydrocarbons [27]
C–H bending (overtone)	1326	O	1324	1326	O		CH_2_ groups
C–O stretching	O	O	1260 (shoulder)	1260 (shoulder)	1257 (shoulder)	1270–1260	Biphenyl ether C–O [28] / alkyl–phenol C–O [30] / carboxylic acids C–O [39]
	1223	1210	1235; 1223	1234; 1223	1208	1240–1210	Phenolic C–O [5,24,25,26,27,28,30] and ether bond [29]
C–H bending andC–O stretching	1167	O	1168	1168	O	1175–1160	Aromatic C–H in plane deformation [5,24,28]
	1146	1151	1143	1146	1138	1153–1147	Aromatic C–H in plane deformation [26,28] and dimethylerne ether C–O–C– bridges [29]
	1064	1124; 1102 (shoulders)	1077	1077	1098; 1077	1120–1060	Aromatic C–H in plane deformation [5,8,24,28] and dimethylene ether C–O–C [5,26,27]
C–O stretching	1031; 1006	1029; 1005	1012	1035; 1013	1032; 1011	1058–1010	Alcoholic C–O (methylol groups) [5,24,25,26,27,28,29]
C–H bending	O	O	992	992	O	997–960	Phenol with trisubstitution at 1,2,4 positions [25,28] / =C–H def. of aryl and/or a,b–unsaturated carboxylic acid [38]
	O	O	933	932	O	950–920	C–O–C ether alyphatic or aromatic C–O–O–C peroxide [32]; O–H out of plane of carboxylic acids [39]
	884	885	880	881	883	890–875	Phenol with tri–substitution at 1,2,4 positions (*o,p*) [5,13] and tetra–substitution at 1,2,4,6 positions *(o,o',p)* [5,24,25,26,28,30]
	818	828	822	820	822	826–814	Phenol with di–substitutions at 1,4 positions *(p)* [5,24,25,26,28,30] and tri–substitution at 1,2,4 positions *(o,p)* [13]
	779	O	775	778	777	790–780	Phenol with tri–substituions at 1,2,6 positions *(o,o')* [5,24,28]
	759	767	753	754	748	760 –756	Phenol with di–substituions at 1,2 positions *(o)* [5,24,25,28,31] and tri–substituions at 1,2,6 positions [30,31]
	O	O	714	714	O	–	CH_2_ rocking [32]
	685	695	680	680 (shoulder)	700	694–690	Monosubstituted ring [5,24,25,28]

**Table 4:** General changes detected by µ-FTIR on the phenol formaldehyde (PF) foams by comparing the spectra of the *“Unaged”* and *Naturally Aged* open- and closed-cell PF foams.

Bond Type	Wavenumber (cm^−1^)	Change Type	Assignment
C=O stretching	1751	→ shift to 1744	changes in aryl carboxylic acids conformation
	1721	↑ increase	formation of hydroxy acids
	1650	↑ increase	formation of benzophenone through oxidation of methylene bridges
C–H bending	1447; 1326	↓ decrease	decrease of methylene bridge through primary oxidation
O–H in plane bending	1354	↓ decrease	slight reduction of phenolic O–H through secondary oxidation
C–H bending and C–O stretching	1168–1064	↓ decrease	ether bridge oxidation andvariation of aromatic C–H conformation
C–O stretching	1005–1035	↓ decrease	decrease of methylol groups

**Table 5:** pH values and their standard deviations registered for the *“Unaged”* and *Naturally Aged* open- and closed-cell type of phenol formaldehyde (PF) foams.

Structure Cell Type	Samples		Color	pH
Open-cell	Gardol	“Unaged”		2.8 ± 0.01
		Natually Aged		2.9 ± 0.06
	Oasis	Unaged		2.8 ± 0.00
		Natually Aged		2.9 ± 0.00
Closed-cell	Balsa Foam Soft Density	“Unaged”		3.8 ± 0.00
		Naturally Aged 1		4.0 ± 0.06
		Naturally Aged 2		4.1 ± 0.06
	Balsa Foam 5 PCF	“Unaged”		4.0 ± 0.00
		Naturally Aged 1		4.2 ± 0.00
		Naturally Aged 2		4.3 ± 0.00
		Naturally Aged 3		4.4 ± 0.00
	Austrotherm	“Unaged”		3.0 ± 0.06
		Naturally Aged 1		3.0 ± 0.00
		Naturally Aged 2		3.1 ± 0.01

## Data Availability

The data presented in this study as well as additional data on OM, µ-FTIR, FORS, and pH results are available on request from the corresponding author.
